# Risk factors of cervical insufficiency in women with PCOS undergoing IVF-ET treatment: a case-control study

**DOI:** 10.3389/fendo.2025.1498443

**Published:** 2025-06-26

**Authors:** Xueqing Zhao, Shenglong Ye, Xin Yan, Rong Li, Yongqing Wang

**Affiliations:** ^1^ Department of Obstetrics and Gynecology, Peking University Third Hospital, Beijing, China; ^2^ National Clinical Research Center for Obstetrics and Gynecology, Peking University Third Hospital, Beijing, China; ^3^ State Key Laboratory of Female Fertility Promotion, Peking University Third Hospital, Beijing, China; ^4^ Key Laboratory of Assisted Reproduction (Peking University), Ministry of Education, Beijing, China; ^5^ Beijing Key Laboratory of Reproductive Endocrinology and Assisted Reproductive Technology, Beijing, China; ^6^ Department of Obstetrics and Gynaecology, Peking University People’s Hospital, Beijing, China; ^7^ Beijing Obstetrics and Gynecology Hospital, Capital Medical University. Beijing Maternal and Child Health Care Hospital, Beijing, China

**Keywords:** cervical insufficiency, polycystic ovarian syndrome, IVF-ET, risk factor, preterm (birth), abortion

## Abstract

**Objective:**

This study aims to investigate the risk factors associated with cervical insufficiency (CI) in women with polycystic ovarian syndrome (PCOS) who have undergone *in vitro* fertilization and embryo transfer (IVF-ET) treatment.

**Methods:**

This study included 746 women diagnosed with PCOS. These women successfully achieved pregnancy through IVF-ET between 2016 and 2023 and subsequently delivered at Peking University Third Hospital. Women were categorized into a study group and a control group based on the presence of CI. Additionally, stratified analyses were performed within each group according to whether fresh or frozen embryos were used for transfer, to investigate the clinical characteristics and risk factors associated with CI.

**Results:**

In women with PCOS undergoing IVF-ET, BMI (OR=1.195, 95% CI: 1.043-1.290, P<0.001), AMH (OR=1.158, 95% CI: 1.092-1.227, P<0.001), frequency of hysteroscopy operations (OR=1.587, 95% CI: 1.202-2.094, P=0.001), prior gravidity (OR=1.956, 95% CI: 1.459-2.621, P<0.001), and occurrence of twin pregnancies (OR=3.028, 95% CI: 1.563-5.865, P=0.001) were found to be positively associated with the incidence of CI. The cut-off value of BMI and AMH were respectively 22.25kg/m^2^ and 9.965ng/ml. Different IVF-ET protocols and hysteroscopic operation within 6 months before pregnancy is not a risk factor for CI. Among women with PCOS undergoing fresh embryo transfer, elevated androstenedione levels (OR=3.250, 95% CI: 1.129-9.359, P=0.029) were identified as a risk factor for CI. In contrast, for women with PCOS undergoing frozen embryo transfer, multiple factors including BMI (OR=1.254, 95% CI: 1.134-1.388, P<0.001), AMH levels (OR=1.232, 95% CI: 1.144-1.327, P<0.001), frequency of hysteroscopy procedures (OR=1.603, 95% CI: 1.155-2.224, P=0.005) and prior gravidity (OR=2.423, 95% CI: 1.674-3.508, P<0.001), were associated with an increased risk of CI.

**Conclusion:**

The study analysed risk factors for cervical insufficiency in women with PCOS who underwent IVF-ET treatment, such as BMI, AMH, frequency of hysteroscopy operations, prior gravidity, twin pregnancy. Providing a basis for the identification and prediction of people at high risk of cervical insufficiency.

## 1 Introduction

Cervical insufficiency (CI) manifests as the flattening, thinning, dilation, and widening of the cervical canal before reaching full-term pregnancy, often resulting in miscarriage, stillbirth or preterm birth (1). With an incidence rate estimated at approximately 1%–3% (1, 2), CI stands as a significant contributor to miscarriage, still birth or preterm birth.

Polycystic ovarian syndrome (PCOS) is an endocrine disorder distinguished by hyperandrogenemia and insulin resistance. The prevalence of PCOS among Chinese women of childbearing age is approximately 5.6% (3). The Rotterdam criteria (4) serve as the internationally endorsed diagnostic framework for PCOS and are widely adopted globally. Clinical presentations of PCOS typically encompass irregular menstruation or amenorrhea, infertility, elevated androgen levels, and obesity.

In a previous study, it was observed that the incidence of CI is higher among women with PCOS compared to those without PCOS (5, 6). However, investigations into the specific risk factors associated with CI among women with PCOS have been limited. Comprehensive assessment of endocrine levels in patients undergoing IVF-ET before pregnancy offers valuable insights for analysis. Since different embryo transfer protocols may result in altered sex hormone levels, our study also focused on the effect of different embryo transfer protocols on the incidence of CI. Our research enables the earlier identification of women with PCOS at high risk of CI, thereby informing and enhancing their clinical management.

## 2 Materials and methods

### 2.1 Study cohort

In this retrospective observational study, data were extracted from the hospital’s electronic medical record system, with diagnoses confirmed by clinicians. Our cohort comprised 746 women diagnosed with PCOS who achieved pregnancy following IVF-ET treatment at Peking University Third Hospital and subsequently delivered there between 2016 and 2023.

In all the study populations, we excluded patients with a history of cervical surgery, such as cervical cold-knife conization(CKC), cervical loop electrosurgical excision procedure(LEEP), cervical excision, and congenital abnormal of cervix, unicornous uterus, didelphic uterus, bicornuate uterus, septate uterus,arcuate uterus.

### 2.2 Study endpoints

Currently, the diagnostic criteria (1, 2, 7) for CI primarily encompass medical history, physical examination, or ultrasonic evaluation during pregnancy, along with cervical function testing before pregnancy. Medical history assessment predominantly focuses on painless cervical dilatation or shortening in the second or third trimester, leading to miscarriage or preterm delivery. During pregnancy, apart from signs of infection, bleeding, or uterine contractions, CI should be considered if physical examination reveals cervical dilation, or if ultrasound indicates cervical length ≤25mm with progressive shortening before the 24th week of pregnancy. Pre-pregnancy cervical function testing involves the passage of a size 8 Hegar dilator through the cervical orifice without resistance.

### 2.3 Data collection

The data collected in this study encompassed various factors, including age; pre-pregnancy BMI (weight (kg)/height² (m²)); levels of hormones such as follicle stimulating hormone (FSH), luteinizing hormone (LH), estradiol (E2), testosterone (T), androstenedione (A), and anti-Müllerian hormone (AMH); type of embryo transfer cycle(fresh or frozen); protocol for controlled ovarian stimulation or endometrial preparation, including ultralong GnRH (gonadotropin-releasing hormone) agonist, long GnRH agonist, short GnRH agonist, and GnRH agonist in fresh embryo cycles, as well as natural or artificial cycles in frozen embryo cycles; frequency of hysteroscopy operations and curettages (total number of such interventions performed during the pre-pregnancy period); whether the last hysteroscopy operation occurred less than six months from the last menstruation; previous gravidity and para; occurrence of twin pregnancies; pre-pregnancy hypertension; prepregnancy diabetes; and medical history. Because women with PCOS have irregular menstrual cycles, levels of hormones are tested at the initial appointment, which can be any day of the menstrual cycle.

### 2.4 Data analysis

All data were retrieved from the electronic medical record system of Peking University Third Hospital. Microsoft Excel 2020 was utilized for data management, while statistical analyses were conducted using Statistical Package for the Social Sciences version 27.0 (SPSS). Descriptive statistics were employed, with measurement data presented as mean ± standard deviation (x ± s) for normally distributed data and as median and quartile [M (P25, P75)] for non-normally distributed data, utilizing t-tests and rank sum tests, respectively, for inter-group comparisons. Categorical variables were expressed as sample number (%) and assessed using the chi-square test or Fisher exact probability method for inter-group comparisons.

Logistic regression was employed to analyze independent variables with CI as the outcome of interest. In the initial analysis, we conducted univariate logistic regression to evaluate the association between each potential risk factor and the risk of CI occurrence, setting the significance level at p < 0.05. Based on the univariate analysis results, we incorporated significant independent variables (p < 0.05) into a multivariate logistic regression model to further assess the independent effects of these factors while adjusting for potential confounders. To ensure model robustness, we employed forward stepwise selection for variable inclusion. All tests were two-sided, with a significance level set at P<0.05.

## 3 Results

### 3.1 Characteristics of the study population

**Figure 1:**
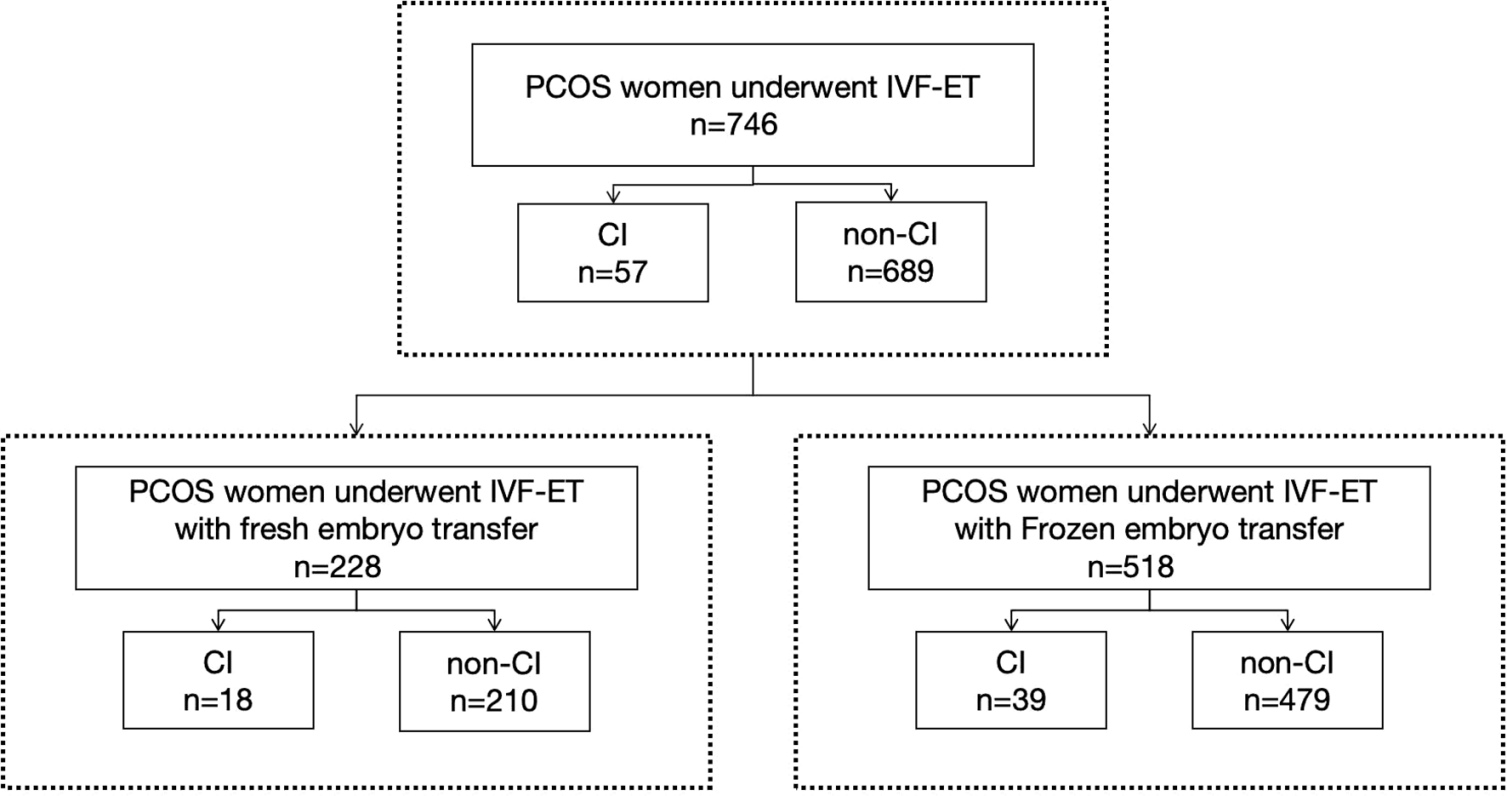
This study included 746 women with PCOS who received IVF-ET treatment, of which 228 received fresh cycle embryo transfer and 518 received frozen cycle embryo transfer.

**Figure 2:**
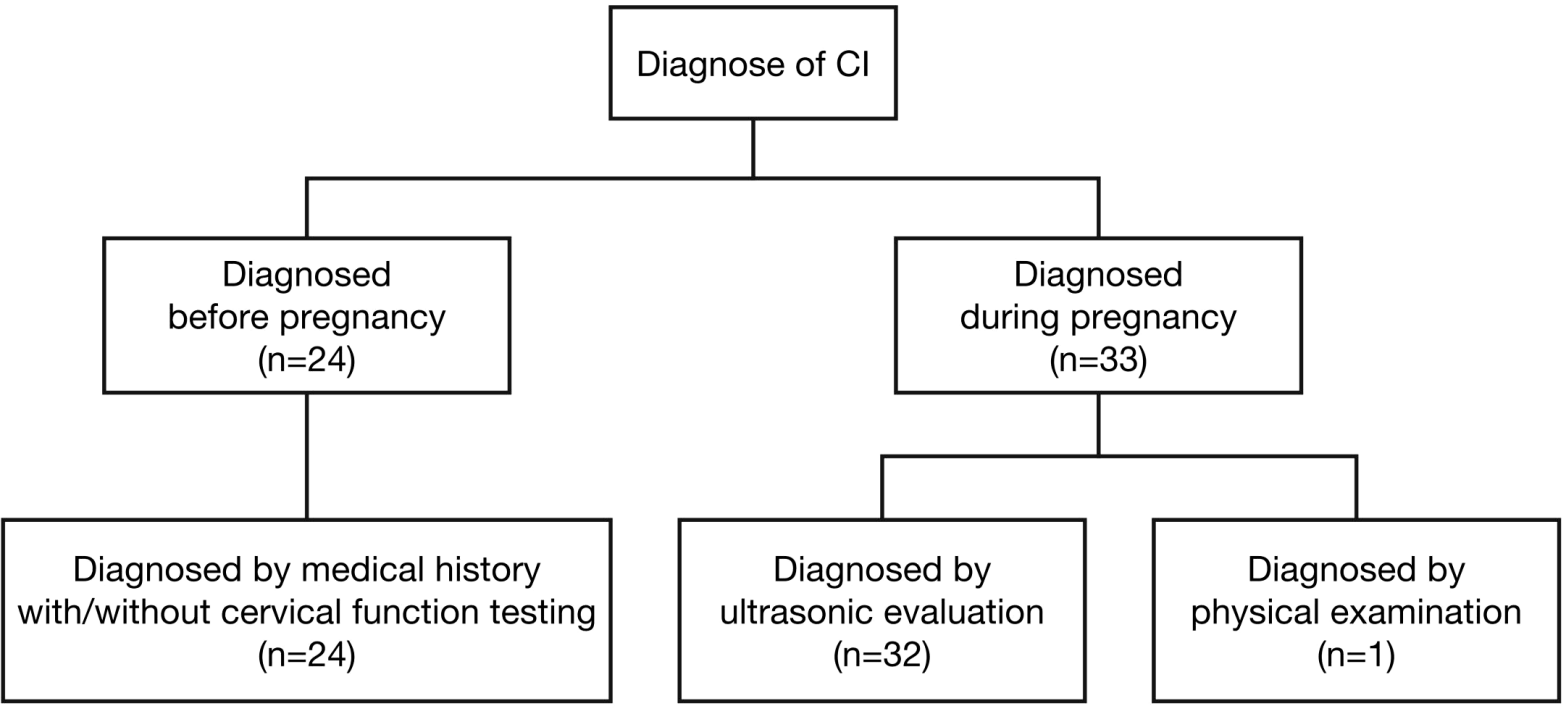
In women with PCOS who received IVF-ET treatment, 57 women were diagnosed as CI, among them 24 women were diagnosed before pregnancy based on medical history with or without cervical function testing and 33 women were diagnosed during pregnancy, including 32 women based on ultrasonic evaluation and one woman based on physical examination.

Among the 746 PCOS cases undergoing IVF-ET, 57 were diagnosed with CI, resulting in an incidence rate of 7.6%, shown as **Figure 1**. Among them 24 women were diagnosed before pregnancy based on medical history with or without cervical function testing and 33 women were diagnosed during pregnancy, including 32 women based on ultrasonic evaluation and 1 woman based on physical examination. The diagnosis of CI in our study population is shown in **Figure 2**. In this study, a total of 228 women underwent fresh embryo transfer, among whom 19 were diagnosed with CI; 518 women underwent frozen embryo transfer, among whom 39 were diagnosed with CI. The specific distribution is shown in **Figure 1**.

In the entire study population, the median age was 32 years, the median BMI was 24.80 kg/m², the median androstenedione level was 7.95 nmol/L, and the median AMH level was 6.46 ng/ml. Among the participants, 17.2% had androstenedione levels exceeding 2.53 nmol/L, 20.5% was twin pregnancy, 3.2% had prepregnancy diabetes, and 5.1% had prepregnancy hypertension.

### 3.2 Risk factors of CI in PCOS cases undergoing IVF-ET

In comparison to non-CI cases, those with CI exhibited significantly higher values in BMI (25.45 ± 3.81 vs. 24.70 (20.45, 25.40)), AMH (8.75 (4.66, 14.01) vs. 6.41 (4.08, 9.14)), frequency of hysteroscopy operations, frequency of curettages, the proportion of cases undergoing hysteroscopy operations within six months before pregnancy (22.8% vs. 16.0%), frequency of previous gravida, proportion of twin pregnancies (31.6% vs. 19.6%), and proportion of hypertension (14.0% vs. 4.4%). Refer to **Table 1** for detailed comparisons.

**Table 1:** Characteristics in women with PCOS with and without CI.

	Total (n=746)	CI (n=57)	Non-CI (n=689)	p*
Age, y	32 (30,35)	32.53 ± 3.33	32 (30,35)	0.539
BMI, kg/m^2^	24.80 (20.60,25.00)	25.45 ± 3.81	24.70 (20.45,25.40)	<0.001
FSH, mIU/ml	6.11 (4.99,7.62)	6.03 (4.81,7.35)	6.12 (4.99,7,64)	0.547
E2, pmol/L	167.00 (126.88,297.00)	157.00 (127.25,223.50)	167.50 (126.75,302.25)	0.209
LH, mIU/ml	4.56 (2.88,6.89)	4.29 (2.90,6.54)	4.56 (2.88,6.95)	0.679
T, nmol/Lnormal				
>2.53	128 (17.2%)	14 (24.6%)	114 (16.5%)	0.123
≤2.53	618 (82.8%)	43 (75.4%)	575 (83.5%)	
A, nmol/L	7.95 (5.67,12.23)	10.32 ± 5.26	7.91 (5.67,12.00)	0.087
AMH, ng/ml	6.46 (4.17,9.46)	8.75 (4.66,14.01)	6.41 (4.08,9.14)	0.004
Embryo transfer cycle				
Fresh	228 (30.6%)	18 (31.6%)	210 (30.5%)	0.862
Frozen	518 (69.4%)	39 (68.4%)	479 (69.5%)	
Hysteroscopy operation, times				
0	389 (52.1%)	19 (33.3%)	370 (53.7%)	<0.001
1	290 (38.9%)	23 (40.4%)	267 (38.8%)	
2	50 (6.7%)	11 (19.3%)	39 (5.7%)	
3	12 (1.6%)	2 (3.5%)	10 (1.5%)	
≥4	5 (0.7%)	2 (1.8%)	3 (0.4%)	
Curettage,times				
0	564 (75.6%)	37 (64.9%)	527 (76.3%)	<0.001
1	143 (19.2%)	16 (28.1%)	127 (18.6%)	
2	32 (4.3%)	1 (1.8%)	31 (4.5%)	
3	7 (0.9%)	3 (5.3%)	4 (0.6%)	
Last hysteroscopy operation within 6 months from the last menstruation				
≤6months	123 (16.5%)	13 (22.8%)	110 (16.0%)	0.013
>6months	234 (31.4%)	25 (43.9%)	209 (53.7%)	
No operation	389 (52.1%)	19 (33.3%)	370 (53.7%)	
Gravidity, times				
0	463 (62.1%)	19 (33.3%)	444 (64.4%)	<0.001
1	188 (25.2%)	25 (43.9%)	163 (23.7%)	
2	72 (9.7%)	10 (17.5%)	62 (9.0%)	
3	18 (2.4%)	2 (3.5%)	16 (2.3%)	
≥4	5 (0.7%)	1 (1.8%)	4 (0.6%)	
Para, times				
0	708 (96.8%)	56 (98.2%)	652 (94.6%)	0.233
1	24 (3.2%)	1 (1.8%)	37 (5.4%)	
Twin pregnancy	153 (20.5%)	18 (31.6%)	135 (19.6%)	0.031
Diabetes	24 (3.2%)	4 (7.0%)	20 (2.9%)	0.091
Hypertension	38 (5.1%)	8 (14.0%)	30 (4.4%)	0.001

BMI, body mass index; FSH, follicle stimulating hormone; LH, luteinizing hormone; E2, estradiol; T, testosterone; A, androstenedione; AMH anti-Müllerian hormone; p* value is the result obtained from the statistical analysis of the CI group and the non-CI group.

Univariate-factor analysis revealed that BMI, AMH, frequency of hysteroscopy operations, frequency of curettages, last hysteroscopy operation within 6 months before pregnancy, frequency of previous gravida, occurrence of twin pregnancies, and presence of hypertension exhibited positive correlations with CI, as illustrated in **Table 2**.

**Table 2:** Univariate and multiple factor regression analysis of women with PCOS.

	Univariate factor regression				Multiply factor regression			
	P	OR	95%CI		P	OR	95%CI	
			Down	Up			Down	Up
Age, y	0.630	1.020	0.941	1.106				
BMI, kg/m^2^	<0.001	1.152	1.079	1.231	<0.001	1.195	1.043	1.290
FSH, mIU/ml	0.613	0.975	0.885	1.075				
E2, pmol/L	0.118	1.000	1.000	1.000				
LH, mIU/ml	0.903	0.996	0.933	1.064				
T>2.53, nmol/L	0.126	1.642	0.870	3.101				
A, nmol/L	0.148	1.035	0.988	1.085				
AMH, ng/ml	<0.001	1.119	1.061	1.181	<0.001	1.158	1.092	1.227
Embryo transfer cycle	0.862	0.950	0.531	1.699				
Hysteroscopy operation, times	<0.001	1.734	1.340	2.243	0.001	1.587	1.202	2.094
Curettage,times	0.029	1.518	1.043	2.208				
Hysteroscopy operation and curettage, times	<0.001	1.615	1.320	1.976				
Last hysteroscopy operation within 6 months	0.009	1.572	1.118	2.209				
Gravidity, times	<0.001	1.682	1.298	2.178	<0.001	1.956	1.459	2.621
Para, times	0.258	0.315	0.042	2.337				
Twin pregnancy	0.034	1.894	1.051	3.414	0.001	3.028	1.563	5.865
Diabetes	0.102	2.525	0.833	7.655				
Hypertension	0.003	3.586	1.561	8.242				

BMI, body mass index; FSH, follicle stimulating hormone; LH, luteinizing hormone; E2, estradiol; T, testosterone; A, androstenedione; AMH anti-Müllerian hormone.

After adjusting for the factors with P < 0.05 in the above univariate analysis, multifactorial analysis revealed that BMI (OR=1.195, 95% CI: 1.043–1.290, P<0.001), AMH (OR=1.158, 95% CI: 1.092–1.227, P<0.001), frequency of hysteroscopy operations (OR=1.587, 95% CI: 1.202–2.094, P=0.001), frequency of previous gravida (OR=1.956, 95% CI: 1.459–2.621, P<0.001), and occurrence of twin pregnancies (OR=3.028, 95% CI: 1.563–5.865, P=0.001) demonstrated a positive correlation with CI. In women with PCOS underwent IVF-ET treatment, neither testosterone>2.53nmol/L nor androstenedione were associated with the incidence of CI, as depicted in **Table 2**.

We used the ROC curve to calculate the cut-off value of BMI and AMH levels in identifying CI. The cut-off value of BMI and AMH were respectively 22.25kg/m^2^ and 9.965ng/ml. The cuttoff value for BMI had a sensitivity of 82.5% and a specificity of 44.8% and a sensitivity of 45.6% and a specificity of 79.7% for the cuttoff value of AMH.

### 3.3 Risk factors of CI in PCOS cases undergoing different type of embryo transfer

In the study population undergoing frozen embryo transfer, several variables exhibited significant differences between CI and non-CI cases. Specifically, BMI (25.60 ± 3.73 vs. 22.30(20.30, 24.80)), AMH levels (11.38 ± 5.90 vs. 6.80(4.50, 14.11)), utilization of artificial cycle (97.4% vs. 81.8%), frequency of hysteroscopy operations, frequency of curettages, proportion of cases undergoing hysteroscopy operations within six months before pregnancy (20.5% vs. 15.7%), frequency of previous gravida, and prevalence of prepregnancy hypertension (20.5% vs. 5.4%) were higher in CI cases compared to non-CI cases, as illustrated in **Table 3**.

**Table 3:** Characteristics in women with PCOS who underwent fresh and frozen embryo transfer.

	Fresh embryo transfer			Frozen embryo transfer		
	CI (n=18)	Non-CI (n=210)	P	CI (n=39)	Non-CI (n=479)	P
Age, y	32.38 ± 2.68	32.00 (30.00,35.00)	0.954	32.97 ± 3.39	32.00 (30.00,35.00)	0.407
BMI, kg/m^2^	25.12 ± 4.06	23.60 (21.23,26.70)	0.322	25.60 ± 3.73	22.30 (20.30,24.80)	<0.001
FSH, mIU/ml	6.16 (4.22,8.59)	6.42 (5.01,8.91)	0.685	5.85 (4.87,6.87)	5.93 (4.97,7.42)	0.664
E2, pmol/L	142.53 (100.55,171.25)	144.00 (112.68,176.63)	0.829	169.00 (129.50,307.00)	190.00 (127.50,457.50)	0.155
LH, mIU/ml	4.77 ± 2.85	4.15 (2.76,6.19)	0.939	4.63 (2.82,6.49)	4.80 (2.89,7.34)	0.583
T, nmol/L						
>2.53	6 (33.3%)	28 (13.3%)	0.022	8 (20.5%)	86 (18.0%)	0.690
≤2.53	12 (66.7%)	182 (86.7%)		31 (79.5%)	393 (82.0%)	
A, nmol/L	7.84 (6.73,11.95)	7.53 (5.45,11.20)	0.265	10.73 ± 5.79	8.05 (5.71,12.70)	0.166
AMH, ng/ml	4.53 (2.65,6.54)	5.36 (3.31,7,54)	0.440	11.38 ± 5.90	6.80 (4.50,14.11)	<0.001
Protocol of controlled ovarian stimulation/Endometrial preparation plan						
Short agonist	1 (5.6%)	1 (0.5%)	0.124			
Long agonist	2 (11.1%)	14 (6.7%)				
Ultralong agonist	2 (11.1%)	18 (8.6%)				
GnRH agonist	13 (72.2%)	177 (84.3%)				
Artificial cycle				38 (97.4%)	392 (81.8%)	0.013
Natural cycle				1 (2.6%)	87 (18.2%)	
Hysteroscopy operation, times						
0	8 (44.4%)	124 (59.0%)	0.124	11 (28.2%)	246 (51.4%)	<0.001
1	7 (38.9%)	74 (35.2%)		16 (41.0%)	193 (40.3%)	
2	3 (16.7%)	9 (4.3%)		8 (20.5%)	30 (6.3%)	
3	0	3 (1.4%)		2 (5.1%)	7 (1.5%)	
≥4	NA.	NA.		2 (5.1%)	3 (0.6%)	
Curettage,times						
0	13 (72.2%)	171 (81.4%)	0.100	24 (61.5%)	356 (74.3%)	0.013
1	4 (22.2%)	31 (14.8%)		12 (30.8%)	96 (20.0%)	
2	0 (0.00%)	7 (3.3%)		1 (2.6%)	24 (5.0%)	
3	1 (5.6%)	1 (0.5%)		2 (5.1%)	3 (0.6%)	
Last hysteroscopy operation within 6 months from the last menstruation						
≤6months	5 (27.8%)	35 (16.7%)	0.395	8 (20.5%)	75 (15.7%)	0.019
>6months	5 (27.8%)	51 (24.3%)		10 (51.3%)	158 (33.0%)	
No operation	8 (44.4%)	124 (59.0%)		11 (28.2%)	246 (51.4%)	
Gravidity, times						
0	10 (55.6%)	158 (75.3%)	0.001	9 (23.1%)	286 (59.7%)	<0.001
1	7 (38.9%)	35 (16.7%)		18 (46.2%)	128 (26.7%)	
2	0 (0.00%)	13 (6.2%)		10 (25.6%)	49 (10.2%)	
≥3	1 (5.6%)	4 (1.9%)		2 (5.1%)	16 (3.3%)	
Para, times						
0	18 (100.0%)	205 (97.6%)	0.508	38 (97.4%)	447 (93.3%)	0.311
1	0 (0.00%)	5 (2.4%)		1 (2.6%)	32 (6.7%)	
Twin pregnancy	8 (44.4%)	64 (30.5%)	0.221	10 (25.6%)	71 (85.2%)	0.074
Diabetes	1 (5.6%)	1 (0.5%)	0.027	3 (7.7%)	19 (4.0%)	0.267
Hypertension	0 (0.00%)	4 (1.9%)	0.555	8 (20.5%)	26 (5.4%)	<0.001

BMI, body mass index; FSH, follicle stimulating hormone; LH, luteinizing hormone; E2, estradiol; T, testosterone; A, androstenedione; AMH anti-Müllerian hormone.

Univariate-factor analysis revealed that BMI, AMH, utilization of natural cycle, frequency of hysteroscopy operations, last hysteroscopy operation within 6 months before pregnancy, and frequency of previous gravida exhibited a positive correlation with CI. Adjust for the confounding factors of the above variables using multivariate analysis, results revealed that BMI (OR=1.254, 95% CI: 1.134–1.388, P<0.001), AMH (OR=1.232, 95% CI: 1.144–1.327, P<0.001), frequency of hysteroscopy operations (OR=1.603, 95% CI: 1.155–2.224, P=0.005), and frequency of previous gravida (OR=2.423, 95% CI: 1.674–3.508, P<0.001) demonstrated a positive correlation with CI. But testosterone>2.53nmol/L and androstenedione were not risk factors of CI in women with PCOS underwent frozen embryo transfer, as depicted in **Table 4**.

**Table 4:** Univariate and multiply factor regression analysis of women with PCOS underwent fresh and frozen embryo transfer.

	Fresh embryo transfer				Frozen embryo transfer cycle							
	Univariate factor regression				Single factor regression				Multiply factor regression			
	P	OR	95%CI		P	OR	95%CI		P	OR	95%CI	
			Down	Up			Down	Up			Down	Up
Age, y	0.832	0.984	0.848	1.142	0.470	1.036	0.941	1.141				
BMI, kg/m^2^	0.308	1.063	0.945	1.196	<0.001	1.202	1.108	1.304	<0.001	1.254	1.134	1.388
FSH, mIU/ml	0.955	0.996	0.857	1.157	0.527	0.959	0.842	1.092				
E2, pmol/L	0.789	0.999	0.995	1.004	0.116	1.000	1.000	1.000				
LH, mIU/ml	0.876	0.987	0.843	1.157	0.966	0.998	0.929	1.073				
T>2.53, nmol/L	0.029	3.250	1.129	9.359	0.690	1.179	0.524	2.655				
A, nmol/L	0.538	1.029	0.939	1.129	0.180	1.038	0.983	1.096				
AMH, ng/ml	0.944	1.005	0.879	1.149	<0.001	1.163	1.091	1.239	<0.001	1.232	1.144	1.327
Protocol of controlled ovarian stimulation (compared with short agonist)												
Short agonist	0.278											
Long agonist	0.225	0.143	0.006	3.310								
Ultralong agonist	0.169	0.111	0.005	2.550								
GnRH agonist	0.070	0.073	0.004	1.243								
Endometrial preparation plan (compared with artificial cycle)					0.037	0.119	0.016	0.875				
Hysteroscopy operation, times	0.142	1.613	0.853	3.050	<0.001	1.781	1.333	2.379	0.005	1.603	1.155	2.224
Curettage,times	0.240	1.531	0.752	3.113	0.063	1.523	0.978	2.372				
Last hysteroscopy operation within 6 months	0.179	1.490	0.833	2.667	0.025	1.619	1.064	2.464				
Gravidity, times	0.091	1.535	0.935	2.523	<0.001	1.776	1.304	2.420	<0.001	2.423	1.674	3.508
Para, times	/	/	/	/	0.331	0.368	0.049	2.765				
twin pregnancy	0.227	1.825	0.668	4.383	0.078	1.982	0.925	4.244				
Diabetes	0.081	12.294	0.736	205.339	0.276	2.018	0.570	7.141				
Hypertension	/	/	/	/	<0.001	4.496	1.880	10.753				

BMI, body mass index; FSH, follicle stimulating hormone; LH, luteinizing hormone; E2, estradiol; T, testosterone; A, androstenedione; AMH anti-Müllerian hormone.

In fresh cycle embryo transfer, all of CI patients have a previous delivery frequency of 0, and all of CI patients do not have chronic hypertension, so logistic regression analysis cannot show the results of both.

The cut-off value of BMI and AMH were respectively 22.25kg/m^2^ and 9.965ng/ml. The cuttoff value for BMI had a sensitivity of 84.6% and a specificity of 48.9% and a sensitivity of 59.0% and a specificity of 75.6% for the cuttoff value of AMH.

In women with PCOS who undergoing fresh embryo transfers, testosterone>2.53nmol/L (33.3% vs.13.3%), frequency of previous gravida, and prevalence of prepregnancy diabetes (5.6% vs. 0.5%) were higher in CI cases compared to non-CI cases. But only testosterone>2.53nmol/L was positively associated with the incidence of CI and was an independent risk factor for CI (OR=3.250, 95% CI: 1.129–9.359, P=0.029), as depicted in **Table 4**.

## 4 Discussion

Previous studies have shown that women with PCOS have a higher risk of CI (5, 6). This study aims to explore which characteristics of women with PCOS are more likely to develop CI. The results showed that higher BMI, elevated AMH levels, increased frequency of hysteroscopic procedures, prior pregnancy history, and twin pregnancies are significant risk factors for CI in women with PCOS undergoing IVF-ET treatment.

Prior investigations have indicated that a BMI exceeding 30 kg/m² is associated with an increased risk of preterm delivery (8) and BMI > 23.9 kg/m² as a high-risk factor for CI (9). Additionally, a predictive model for CI has identified a BMI > 23.9 kg/m² as a high-risk factor for CI (9). Consistent with these findings, our research has reached similar conclusions, demonstrating a positive correlation between BMI and the incidence of CI. In our study, the proportion of BMI <24 kg/m^2^ (62.9%), BMI ≥24 and <28 kg/m^2^ (24.4%) and BMI >28 kg/m^2^ (12.7%) are similiar to previous study (10), so this part of the population is representative. Furthermore, utilizing BMI as a continuous variable, we observed that for every 1 kg/m² increase in BMI, the incidence of CI increased by a factor of 1.195. We calculated the cut-off value of BMI in predicting CI and found that it was 22.25 kg/m2 in women with PCOS underwent IVF-ET treatment. Possibly due to the rise in BMI, there’s an increase in abdominal pressure, subsequently augmenting the load on the cervix. In addition, obese patients possess distinct physiological and endocrine environments and are susceptible to complications (11), which may contribute to cervical shortening alongside elevated abdominal pressure. Consequently, in clinical practice, weight loss interventions may emerge as the most effective strategy for mitigating the risk of CI in obese PCOS patients.

The cervical remodeling process is characterized by a reduction in collagen content and an elevation in hyaluronic acid levels (12, 13). During the second to third trimesters of pregnancy, a correlation between androgen levels and cervical shortening has been found (14). Androgens may decrease collagen content, potentially contributing to CI (15). Our findings indicated that among women with PCOS undergoing IVF-ET, the proportion of individuals with high testosterone levels before pregnancy was notably elevated in CI cases. However, these differences did not reach statistical significance. But testosterone emerges as an independent risk factor for CI in women with PCOS who undergoing fresh cycle embryo transfer. We hypothesize two potential reasons for these observations: firstly, the limited sample size of fresh cycle embryo transfers may necessitate a larger population for more robust research outcomes. Secondly, despite the lower sensitivity of testosterone compared to androstenedione, the biological activity of free testosterone is considerably stronger (16), potentially leading to more pronounced biological effects and a heightened incidence of CI.

The level of AMH in women with PCOS exceeds that of women without PCOS (17). AMH levels surpassing the 75th percentile are linked to an elevated risk of preterm birth in PCOS patients (18). Given that CI is among the factors contributing to preterm birth, we investigated its potential relationship with CI. Our analysis revealed that in women with PCOS undergoing IVF-ET, AMH served as an independent risk factor for CI. To our knowledge, our study is the first to establish a correlation between AMH levels and CI. We also calculated the cut-off value of AMH in predicting CI and found that it was 9.965 ng/ml. AMH belongs to the transforming growth factor β (TGF-β) superfamily and exerts its effects through AMH receptors. Elevated AMH levels can significantly induce apoptosis in tissues derived from Müllerian tubes (19). AMH receptors has been detected in normal cervical tissues (20). AMH is also involved in extracellular matrix (ECM) formation via the TGF-β signaling pathway. Inhibition of the AMH-receptor binding increases free AMH levels in the serum, reduces the availability of active binding agents, thereby diminishing ECM formation or promoting apoptosis, ultimately leading to CI.

IVF-ET emerges as a potential risk factor for CI. Numerous studies have investigated the pregnancy outcomes associated with various transfer protocols during embryo transfer procedures (21). For instance, Shi Yuhua et al. (22) observed that compared to fresh cycle embryo transfer, frozen cycle embryo transfer reduces the risk of second-trimester pregnancy loss in women with PCOS. Conversely, Liu Y. et al. highlighted a high incidence of CI in cases undergoing frozen cycle embryo transfer (23). Our findings revealed that the incidence of CI in women with PCOS undergoing frozen cycle embryo transfer does not significantly differ from that in those undergoing fresh cycle embryo transfer. Additionally, there appears to be no correlation between different ovulation induction regimens and the incidence of CI in women with PCOS undergoing fresh cycle embryo transfer. Among women with PCOS undergoing frozen cycle embryo transfer, the incidence of CI is higher in artificial cycles compared to natural cycles. Furthermore, univariate analysis indicated an association between protocol of endometrial preparation and CI, although multivariate analysis did not identify it as an independent risk factor for CI.

Previous research has indicated that women who have undergone hysteroscopy within six months prior to pregnancy exhibit a heightened incidence of abortion or preterm delivery (24). In our study, we incorporated data on the number of uterine cavity operations, including hysteroscopy and curettage before pregnancy, as well as the timing of these procedures. Our findings revealed that the number of hysteroscopic surgeries independently contributes to the risk of CI. Additionally, the frequency of curettage correlates positively with the incidence of CI. While hysteroscopy or intrauterine operations performed within six months before pregnancy may increase the risk of CI, they do not emerge as independent risk factors. The mechanical dilation exerted by surgical instruments during hysteroscopy operations may account for why it is identified as an independent risk factor for CI. Currently, hysteroscopy procedures encompass both electrocautery and finer examination scopes, with the latter requiring less cervical dilation. We recommend that all hysteroscopic procedures performed prior to IVF-ET currently employ examination scopes as much as possible to mitigate their impact on cervical function.

In this study, we have for the first time explored the relationship between CI and AMH levels, various IVF-ET protocols, the frequency of hysteroscopy or curettage, and the interval before pregnancy. Our findings have uncovered novel risk factors, thus offering valuable insights for future clinical practice, aiming to furnish a theoretical foundation for the early diagnosis and management of CI in this population.

Considering the retrospective nature of this study, several limitations should be acknowledged. Firstly, the absence of specific data on hysteroscopic surgery poses a challenge in comprehensively assessing its impact on CI, necessitating further detailed research and discussion in this area. Secondly, the specificity of BMI cutoff value and the sensitivity of AMH cutoff value are low. And the relatively small sample size of fresh cycle embryo transfer may introduce bias in the results, warranting larger sample sizes for future research. Lastly, although this study focused on a population receiving IVF-ET, which underwent thorough physical examinations before treatment initiation, future research should explore high-risk factors for CI in other populations.

## 5 Conclusion

Our study conducted a comprehensive analysis of the risk factors associated with CI in PCOS patients undergoing IVF-ET treatment, examining five key aspects: basic demographics, pre-pregnancy endocrine profiles, embryo transfer protocols, history of hysteroscopic surgeries, and medical history/pregnancy-related details. Our findings revealed that high prepregnancy BMI, elevated prepregnancy AMH levels, frequency of hysteroscopy procedures, and occurrence of twin pregnancies and previous pregnancies were identified as independent risk factors for CI. Our study is the first to find a correlation between prepregnancy AMH and CI. Additionally, prepregnancy androgen levels, various IVF-ET protocols, the timing between hysteroscopy or intrauterine procedures and conception, and hypertension may also play contributory roles in CI development, necessitating further research for confirmation.

## Data Availability

The raw data supporting the conclusions of this article will be made available by the authors, without undue reservation.
